# Effects of Milk Polar Lipids on DSS-Induced Colitis Severity Are Dependent on Dietary Fat Content

**DOI:** 10.3390/nu14235145

**Published:** 2022-12-03

**Authors:** Chelsea Garcia, Liya Anto, Christopher N. Blesso

**Affiliations:** Department of Nutritional Sciences, University of Connecticut, Storrs, CT 06269, USA

**Keywords:** colon transcriptome, dietary fat, microbiome, inflammation, dairy, sphingolipids, ceramide, lipidomics

## Abstract

In the United States, over three million adults suffer from inflammatory bowel disease (IBD). The gut microbiome, host immune response, and nutrient-microbial interactions are known to play a role in IBD. The relationship between dairy and IBD is controversial; thus, the objectives of this study were to identify how milk polar lipids (MPLs) and anhydrous milk fat affect colitis disease activity, the colonic transcriptome, and the gut microbiome in a mouse model of chemical-induced colitis. Male and female C57BL/6J mice (n = 120) were randomized into either a low (5% w/w) milk fat or a high (21% w/w) milk fat diet supplemented with either 0%, 1%, or 2% w/w of MPLs for three weeks (n = 10/group/sex). Afterwards, colitis was induced using 1% dextran sodium sulfate in drinking water for five days (colitis induction) and then switched to regular water for five days (colitis recovery). Mice fed added MPLs were protected against colitis when fed a high-fat diet, while added MPLs during low-fat diet attenuated disease activity during the colitis induction period yet promoted colitis and inflammation in male mice during the recovery period. Dietary fat content can alter colitis and influence the anti-inflammatory effect of milk polar lipids.

## 1. Introduction

Inflammatory bowel disease (IBD) comprises Crohn’s disease (CD) and ulcerative colitis (UC), which are diseases that damage and cause inflammation in the epithelial lining of the gastrointestinal tract. During flare-ups, the colon can perforate, resulting in the translocation of bacteria from the intestine into circulation [1]. It is estimated that over three million adults have been diagnosed with IBD in the United States [2], which continues to rise due to the consumption of the Westernized diet [3]. The gut microbiota has become a key factor in IBD risk, including its role in inflammation and its ability to interact with environmental and lifestyle factors [1]. It is well known that the gut microbiome can be modulated by diet and, thus, nutrient-microbial interactions need to be researched in chronic diseases, such as IBD. Many IBD patients will restrict dairy consumption due to its lactose content; however, clinical trials have not supported that the removal of dairy products from the diet improves symptoms for all patients, in fact, there is evidence that it may be beneficial [4,5]. The consumption of dairy is thought to worsen IBD symptoms only in those with a dairy and/or lactose allergy and/or malabsorption [5,6]. Furthermore, a cross-sectional study revealed that the majority of IBD and UC patients that restrict dairy products had more frequent and more extensive disease activity, respectively [7]. Thus, the relationship between dairy products on IBD and inflammation is controversial and needs to be further investigated.

Milk contains milk fat, which is rich in saturated fatty acids and is surrounded by a milk fat globule membrane (MFGM) that is rich in sphingolipids, phospholipids, and MFGM proteins [8]. Milk fat has been shown to exacerbate colitis in interleukin (IL)-10 knockout mice and dextran sodium sulfate (DSS)-treated specific-pathogen-free C57BL/6J mice by promoting the growth of Bilophila wadsworthia via taurine conjugation of hepatic bile acids [9]. In contrast, milk sphingomyelin, a component of milk polar lipids (MPLs), has been shown to be protective against colitis disease activity [10]. There is evidence that MPLs have anti-inflammatory properties and the ability to improve microbial diversity in murine models of high-fat diet induced cardiometabolic disease [11,12,13,14,15]. In addition, mono-colonization of germ-free mice with a strain of sphingolipid-deficient Bacteroides (B.) thetaiotaomicron results in intestinal inflammation and modulation of ceramide metabolites [16]. Interestingly, Bacteroides-derived sphingolipids were lower in those with IBD and negatively correlated with inflammation and host sphingolipid metabolism [16]. Thus, exogenous sphingolipids may be important for controlling colon inflammation; however, the effects of MPLs, as a source of milk sphingolipids, on colon inflammation, and their interactions with milk fat are unclear. Therefore, we sought to understand the dose-dependent effects of MPLs in combination with low (5% w/w) (LFD) or high (21% w/w) (HFD) milk fat diets on DSS-induced colitis severity, colon transcriptomics, fecal lipidomics, and cecal microbial composition and diversity in male and female mice. We hypothesized that anhydrous milk fat (AMF) would worsen colitis disease activity and induce gut dysbiosis, while MPLs would attenuate the HFD effects via their anti-inflammatory and microbiome-modulating capacities.

## 2. Materials and Methods

### 2.1. Animals and Diets

**Figure 1:**
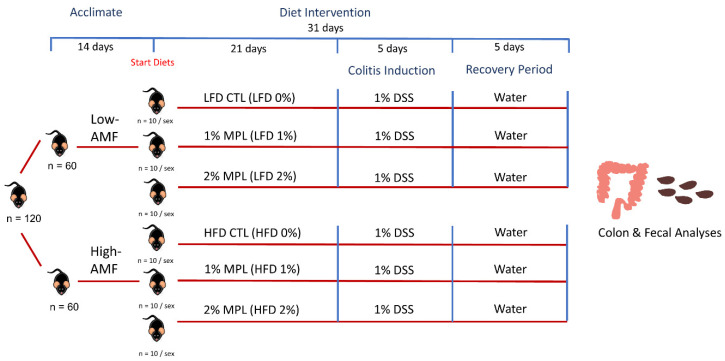
Study Design of Diet Intervention and Colitis Protocol. Male and female C57BL/6J mice (n = 120) were randomized into either a low (5% w/w) milk fat or high (21% w/w) milk fat diet supplemented with either 0%, 1%, or 2% w/w of milk polar lipids (MPL) for three weeks (n = 10/group/sex). Colitis was induced using 1% dextran sodium sulfate (DSS) in drinking water for the next five days and regular drinking water was given for the final five days. Colons were collected for transcriptomics by RNA sequencing, and real-time qRT-PCR, and cecal feces were collected for 16S rRNA sequencing for microbiota analysis. Fecal samples were collected for lipidomics analysis. Disease Activity Index (DAI) was recorded daily consisting of a weight loss, bleeding, and stool consistency score. Abbreviations: AMF, anhydrous milk fat; CTL, control.

Male and female C57BL/6 mice (n = 120) were obtained from Jackson Laboratory (Bar Harbor, ME, USA) and allowed to acclimate for 2 weeks. All mice were housed in a temperature-controlled room and maintained in a 14-h light/10-h dark cycle at the University of Connecticut-Storrs vivarium. All animal experiments were in accordance with the Guide for the Care and Use of Laboratory Animals published by the National Institutes of Health [17]. To investigate the effects of dietary milk fats on colitis severity and the microbiome, we fed the mice a low (5% w/w) milk fat diet or high (21% w/w) milk fat diet with 0%, 1% or 2% w/w supplemented MPL for 3 weeks (n = 10/group/sex). MPL contents of diets were supplemented using beta serum powder (BSP2) (Tatua Dairy Cooperative, Morrinsville, New Zealand) and corresponded to approximately 0.2% (w/w) milk sphingomyelin and 0.4% (w/w) milk sphingomyelin in the 1% (w/w) and 2% (w/w) MPL supplemented diets. Within each LFD (LFD 0%, 1%, 2%) and HFD (HFD 0%, 1%, 2%) conditions, diets were adjusted to match in energy density, macronutrient composition, lactose, calcium, and sodium content. Diet and nutrient compositions are described in Table 1 and Table 2. After the 3 weeks, the mice were given 1% (w/v) DSS in drinking water for 5 days to induce colitis and then given regular drinking water for the last 5 days while remaining on the diet for a recovery period. Administration of 1% (w/v) DSS in drinking water was chosen after a pilot study to determine the optimal dose to induce colitis but not alter the survival rate. During the last 10 days, disease activity index (DAI) scores were recorded daily. The experimental design is represented in Figure 1. On day 10 of the DSS protocol, the mice were fasted for 6 to 8 h prior to being anesthetized with ketamine/xylazine (100 mg/kg ketamine and 10 mg/kg xylazine), followed by blood collection by cardiac puncture following euthanasia. Animals were perfused with sterile saline to clear any residual blood from tissues. Fecal samples were collected and snap-frozen before storage at −80 °C. Tissues were harvested, weighed, snap-frozen in liquid nitrogen, and then stored at −80 °C. Colon length was also measured from the ileocecal valve to the end of the rectum. At the time of sacrifice, colons were collected and used for transcriptomics via RNA-sequencing and real-time quantitative reverse transcriptase polymerase chain reaction (qRT-PCR). Cecal feces and fecal pellets were collected for 16S rRNA sequencing for microbiome analysis and metabolomics analysis, respectively.

### 2.2. Disease Activity Index

DAI was composed of three categories each with its own score of weight loss, stool consistency, and bleeding following the methods described by Kim et al. [18]. DAI scores were used to quantify disease severity as well as serve as an indicator for ethical euthanasia (total score over 3). Stool consistency and bleeding have scores of 0 (normal stool or negative hemoccult), 2 (loose stool or positive hemoccult), or 4 (diarrhea or gross bleeding). While the weight loss category can have a score of 0 (none), 1 (0–10%), 2 (10–15%), 3 (15–20%), and 4 (>20%), which requires ethical euthanasia. DAI scores were collected daily during the colitis induction and recovery period. Both average DAI scores over time and total area under the curve (AUC) of DAI scores are reported.

### 2.3. Colon RNA Isolation, cDNA Synthesis, and Real-Time Quantitative Reverse Transcriptase Polymerase Chain Reaction 

Total RNA was isolated from snap-frozen colon (n = 7/group/sex) using TRIzol (Life Technologies, Carlsbad, CA, USA). Total RNA was treated with DNase I and reverse transcribed using iScript cDNA synthesis kit (Bio-Rad, Hercules, CA, USA). Real-time qRT-PCR was performed using Ssso Advanced SYBR Green Supermix (Bio-Rad) on a CFX96 real-time-PCR detection system (Bio-Rad). Gene expression was normalized to the geometric mean of the reference genes, glyceraldehyde 3-phosphate dehydrogenase (Gapdh), β-actin (Actb), and ribosomal protein, large, P0 (Rplp0) for colon using the 2^−ΔΔCt^ method. See Table S1 for primer sequences used.

### 2.4. Transcriptomics Analysis of Mouse Colon Tissue

RNA was isolated from snap-frozen colon tissues from mice fed the control and 2% MPL groups (n = 4/group/sex). For transcriptome analysis, sample preparation and library constructions were performed at the UConn Center for Genome Innovation (CGI) and then sent to Psomagen, Inc. (Rockville, MD, USA) for next-generation sequencing (RNA-Seq) as previously described [19]. Bioinformatics analysis on RNA-Seq data was conducted at the UConn Computational Biology Core as described [19]. Differential expression analysis was conducted using DESeq2. Adjusted *p*-values were calculated to account for multiple testing by Benjamini-Hochberg method. Gene set enrichment analysis was then conducted using gProfiler (https://biit.cs.ut.ee/gprofiler/gost (accessed on 26 September 2022)) to examine gene ontology.

### 2.5. Gut Microbiota Analysis

Cecal feces samples were aseptically harvested from separately housed mice (n = 4/group/sex) and submitted to the University of Connecticut-Storrs Microbial Analysis, Resources and Services facility for microbiota characterization using 16S V4 analysis as previously described [20]. Following DNA extraction and amplification, PCR products were pooled for quantification, normalized, and cleaned prior to clustering into operational taxonomic units (OTUs) [20].

### 2.6. Fecal Lipidomics Analysis

Fecal samples were collected on day 10 of the DSS protocol for lipidomics analysis conducted by West Coast Metabolomics Center at the University of California-Davis. The organic phase was extracted from fecal samples using the Matyash protocol and used for subsequent lipidomics determination using mass spectrometry. Twenty microliters of sample were extracted using the Matyash extraction procedure which includes MTBE, MeOH, and H2O. The organic (upper) phase was dried down and submitted for resuspension and injection onto the LC, while the aqueous (bottom) phase was dried down and submitted to derivatization for GC. Dried lipid extracts are resuspended with 110 uL of a solution of 9:1 methanol: toluene and 50 ng/mL CUDA and shaken for 20 s, sonicated for 5 min at room temperature, and then centrifuged for 2 min at 16,100 rcf. The samples are then aliquoted into three parts, 33 uL are aliquoted into a vial with a 50 uL glass insert for positive and negative mode lipidomics, and the last is aliquoted into an eppendorf tube to be used as a pool. The samples are then loaded up on an Agilent 1290 Infinity LC stack. The positive mode was run on an Agilent 6530 with a scan range of m/z 120–1200 Da with an acquisition speed of 2 spectra/s. Positive mode has 1 µL injected onto a Acquity Premier BEH C18 1.7 µm, 2.1 × 50 mm Column. The gradient used is 0 min 15% (B), 0.75 min 30% (B), 0.98 min 48% (B), 4.00 min 82% (B), 4.13–4.50 min 99% (B), and 4.58–5.50 min 15% (B) with a flow rate of 0.8 mL/min. The other sample aliquot was run in negative mode, which was run on Agilent 1290 Infinity LC stack, and injected on the same column, with the same gradient, and using an Agilent 6546. The acquisition rate was 2 spectra/s with a scan range of m/z 60–1200 Da. The mass resolution for the Agilent 6530 is 10,000 for ESI (+) and 30,000 for ESI (-) for the Agilent 6546. An analytical UHPLC column was used for data acquisition and the data was processed using four stages. Lipidomics were processed using MassHunter Qual and Mass Profiler Professional to identify target metabolites, align and filter peaks, and compile a peak list that is used to identify lipid species with LipidBlast library. Masshunter Quant was then used to quantify any missing peaks from the first analysis. Data are represented as peak heats for quantification ion (mz value) at each respective retention time. Raw data is normalized by the total ion chromatogram for known metabolites and an internal standard. Metabolomics analysis was executed using MetaboAnalyst 5.0 (https://www.metaboanalyst.ca/ (accessed on 30 October 2022)) to normalize the data and run a one-way ANOVA, principal component analysis, and partial least squares-discriminant analysis.

### 2.7. Statistical Analysis

For other comparisons, significance was determined using a three-way ANOVA (with sex, dietary fat content, and dietary MPL content as between-subjects factors) with multiple comparisons using the Fisher’s Least Significant Difference post-hoc test with SPSS Statistics, version 28 for Windows (IBM, Armonk, NY, USA). A *p*-value < 0.05 was considered statistically significant. Data are reported as mean ± SEM.

## 3. Results

### 3.1. MPLs Attenuate DSS-Induced Colitis Disease Activity in HFD-Fed Mice, but Exacerbate Disease Activity in LFD-Fed Male Mice

At the end of the study, male mice displayed higher body weight (*p* < 0.001), gonadal adipose tissue weight (*p* < 0.001), and liver weight (*p* < 0.001) compared to female mice (Table 3). Mice fed HFD had increased body weight (*p* < 0.001) and gonadal adipose tissue weight (*p* < 0.001) compared to LFD. No differences in total food intake were seen between any groups. Feeding of both 1% MPL (*p* < 0.027) and 2% MPL (*p* < 0.038) dosages lowered liver weights compared to 0% MPL controls, regardless of sex or fat.

**Figure 2:**
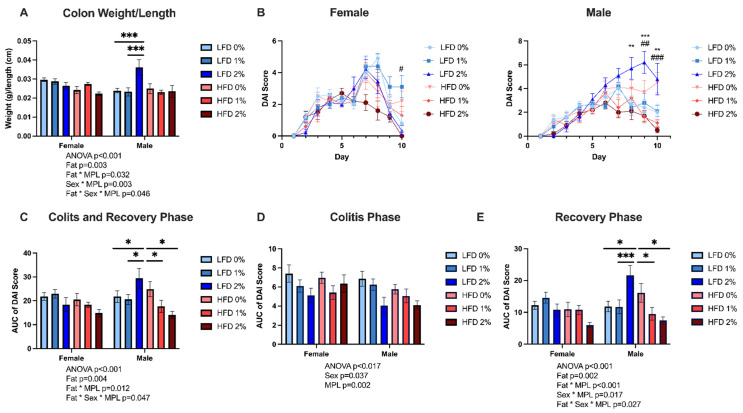
MPLs exacerbate colitis in LFD but attenuate colitis in HFD. (**A**) Colon weight/length ratios. (**B**) Daily DAI scores of female and male mice over the colitis induction and recovery periods. Area under the curve (AUC) of the DAI scores of male and female mice over the 10-day DSS protocol (**C**), and during the colitis induction (**D**), and recovery periods (**E**). For panel B, ** indicates *p*-value < 0.01 for LFD 2% vs. 0% *** indicates *p*-value < 0.001, # indicates *p*-value < 0.05 for HFD 2% vs. 0%, ## indicates *p*-value < 0.01 for HFD 2% vs. 0%, ### *p*-value < 0.001 for HFD 2% vs. 0%. For panels A and C-E, * *p*-value <0.05, *** *p*-value <0.001 using three-way ANOVA with Fisher’s least significant difference for multiple comparisons. Values reported as mean ± standard error of the mean, n = 10/sex/group.

For colon weight: colon length ratios (Figure 2A), where a higher value is reflective of greater inflammation and edema [21], there were significant fat*MPL (*p* = 0.032), sex*MPL (*p* = 0.003), and sex*MPL*fat (*p* = 0.046) interactions. Within males fed 2% MPL, mice fed LFD 2% had increased colon weight: length ratio (*p* < 0.001) compared to HFD 2%, while male mice fed LFD 2% had increased colon weight: length ratio compared to females fed LFD 2% (*p* = 0.002). Male mice fed LFD 2% had increased colon weight: length ratio compared to LFD 1% (*p* < 0.001) and LFD 0% (*p* < 0.001) fed male mice. 

To assess colitis disease severity, a DAI score was determined daily during the colitis induction and recovery periods. By day 2 (Figure 2B) there was a significant main effect of MPL (*p* = 0.005), with mice fed 2% MPL having lower DAI scores than mice fed 1% (*p* = 0.018) and 0% (*p* = 0.002), indicating protection against disease activity during colitis induction. By day 6, the first day of recovery, male mice had higher DAI scores than females (*p* < 0.001). There was a fat*MPL interaction (*p* = 0.004) with mice fed LFD 2% having higher DAI scores than LFD 1% (*p* = 0.004) and LFD 0% (*p* = 0.025). However, mice fed a HFD with 2% had lower DAI scores than HFD 0% (*p* = 0.03). These trends continued throughout the rest of the recovery period, and on the last day of the protocol (day 10), males had higher DAI scores than females (*p* = 0.002) and there were significant fat*MPL (*p* < 0.001), sex*MPL (*p* = 0.003), and fat*sex*MPL (*p* = 0.01) interactions. Male mice fed LFD 2% had higher DAI scores on day 10 compared to male mice fed HFD 2% (*p* < 0.001) and female mice fed LFD 2% (*p* < 0.001). Male mice fed LFD 2% also had higher DAI scores on day 10 than male mice fed LFD 1% (*p* = 0.004) and LFD 0% (*p* = 0.003). In contrast, female mice fed LFD 2% had lower DAI scores on day 10 than female mice fed LFD 1% (*p* = 0.013), but not LFD 0%. Within HFD-fed mice, female mice fed HFD 2% had lower DAI scores on day 10 compared to female mice fed HFD 0% (*p* = 0.018), while in male mice HFD 2% and HFD 1% lowered DAI scores compared to HFD 0% (*p* < 0.001).

When considering DAI scores over the whole 10-day colitis protocol (colitis and recovery period) by calculating the area under the curve (AUC) (Figure 2C), there were significant fat*MPL (*p* = 0.012) and fat*sex*MPL (*p* = 0.047) interactions. Mice fed HFD 2% had lower DAI scores compared to HFD 0% (*p* < 0.001). When also considering sex, male mice fed HFD 2% (*p* = 0.002) and HFD 1% (*p* = 0.037) had lower DAI scores than male mice fed HFD 0%. In contrast, male mice fed LFD 2% had higher DAI scores than LFD 0% males (*p* = 0.027), LFD 1% males (*p* = 0.011), HFD 2% males (*p* < 0.001), and LFD 2% females (*p* = 0.002). Specifically, during the colitis induction period (Figure 2D), female mice had increased DAI scores compared to males (*p* = 0.037), and 2% MPL (*p* < 0.001) and 1% MPL (*p* = 0.043)-fed mice had lower DAI scores than 0% MPL control diets, regardless of sex or dietary fat content. During the recovery period (Figure 2E), there were significant fat*MPL (*p* < 0.001), sex*MPL (*p* =0.017), and fat*sex*MPL (*p* = 0.027) interactions. Male mice fed LFD 2% had higher DAI scores than LFD 1%, LFD 2%, and HFD 2%-fed male mice (*p* < 0.001). In contrast, male mice had lower DAI scores when they were fed HFD 2% (*p* = 0.003) and 1% (*p* = 0.022) compared to when they were fed HFD 0%. The colitis and recovery period scores of the individual components of the DAI (bleeding, stool consistency, and weight loss) are shown in Supplemental Figure S1. MPLs protected against bleeding over the entire 10-day protocol (*p* < 0.001) (Figure S1A). Specifically, mice fed 2% MPLs compared to 0% MPL control-fed mice had less bleeding during the colitis induction (*p* < 0.001), recovery (*p* = 0.002), and combined periods (*p* = 0.002). Male mice lost more weight during the 10-day colitis period, particularly the recovery period (*p* < 0.001), and mice fed LFD 2% lost more weight than mice fed HFD 2% (*p* = 0.002) and LFD 0% (*p* = 0.033) (Figure S1I). During these two periods, mice fed LFD 2% had looser stool (*p* = 0.009) while HFD 2%-fed mice had firmer stool (*p* = 0.007) compared to control-fed mice (Figure S1E). In summary, the DAI scores indicate that during colitis induction, MPL feeding reduced disease activity primarily by reducing bleeding; however, during the recovery period, MPLs worsened disease activity in LFD-fed male mice by increasing weight loss and loose stool, while MPLs attenuated disease severity in HFD-fed male mice by reducing bleeding and loose stool.

### 3.2. MPLs in LFD Exacerbate, While MPLs in HFD Attenuate, Colon Inflammation in Male Mice

**Figure 3:**
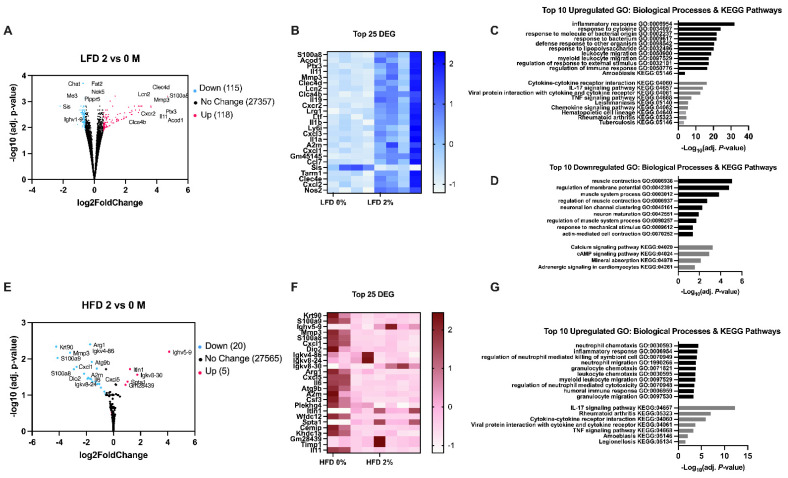
Transcriptomics shows MPLs augment colon inflammatory gene expression in LFD, yet attenuate inflammation in HFD in male mice. (**A**) Volcano plot of LFD 2% vs. 0%-fed male mice (n = 4/group). (**B**) Heat map of top 25 differentially expressed genes (DEG). (**C**) Top 10 upregulated Gene Ontology (GO): Biological Processes and KEGG Pathways. (**D**) Top 10 downregulated GO: Biological Processes and KEGG Pathways. (**E**) Volcano plot of HFD 2% vs. 0%-fed male mice (n = 4/group). (**F**) Heat map of top 25 DEG. (**G**) Top 10 Upregulated GO: Biological Processes and KEGG Pathways.

We conducted transcriptomics analysis through RNA sequencing (RNA-seq) of colon tissue from male and female mice consuming LFD 0%, LFD 2%, HFD 0%, and HFD 2% MPL diets (Table S2). RNA-seq of the colon revealed two-hundred thirty-three differentially expressed genes (DEG) in male mice fed LFD 2% MPL compared to LFD 0% control diet (Figure 3A). A heat map of the top 25 DEG is presented in Figure 3B. Of the DEG, one-hundred eighteen genes were upregulated. Gene set enrichment analysis showed these genes were associated with the inflammatory response, including nuclear factor kappa B (NF-κB) signaling, IL-17 signaling, and tumor necrosis factor (TNF) signaling (e.g., Tradd, Csnk2b, Hsp90ab1, Il17Ra, Akt, Creb3) (Figure 3C). Male mice fed LFD 2% also had increased expression of genes related to cytokine-cytokine receptor interaction, response to other organisms, and innate immune response (e.g., Cxcl5, Tnf, Cxcl1, S100a8, S100a9, Lcn2). The other one-hundred fifteen DEG that were downregulated in male mice fed LFD 2% represented decreased expression of genes related to muscle contraction (e.g., Atb2b2, Atp2b4, Tmod2, Pln) and the neuronal system (e.g., Gnai1, Kcnab1, Kcna1, Glg2) (Figure 3D). The expression of genes involved in calcium (e.g., Atp2b2, Pdgfd, Cacna1e) and cAMP (e.g., Gnai1, Atb2b2, Sstr1) signaling pathways were also reduced in male mice fed LFD 2% males. In contrast, male mice fed HFD 2% had reduced expression of twenty genes related to IL17 signaling, TNF signaling, and cytokine-cytokine receptor interaction (e.g., Mmp3, S100a9, Cxcl1, Cxcl5, Il6) (Figure 3E,G). The top 25 DEG are represented by a heat map in Figure 3F. Only five genes were upregulated in the male mice fed HFD 2% MPLs compared to control (e.g., Ighv5-9, Igkv8-30, Itln1, Spta1, Gm28439) (Figure 3E). There were not any significant DEG in the colon of female mice fed MPLs in either LFD or HFD conditions.

**Figure 4:**
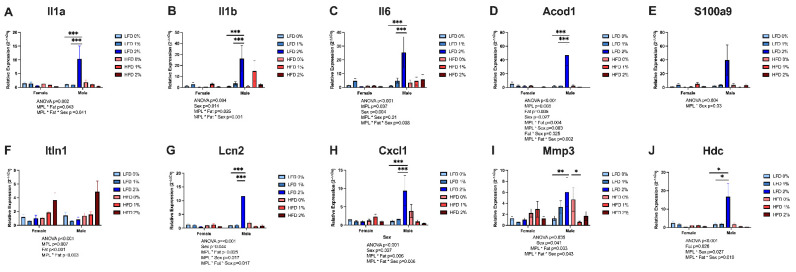
Effect of MPLs on colon gene expression. Colonic RNA gene expression was determined using real-time qRT-PCR and standardized to the geometric mean of Gapdh, Actb (β-actin), and Rplp0 reference genes using the 2^−ΔΔCt^ method. (**A**) Interleukin (IL)-1α (Il1a), (**B**) IL-1β (Il1b), (**C**) IL-6 (Il6), (**D**) aconitate decarboxylase (Acod1), (**E**) S100a9, (**F**) intelectin 1 (Itln1), (**G**) lipocalin 2 (Lcn2), (**H**) CXC motif chemokine receptor 1 (Cxcl1), (**I**) matrix metallopeptidase 3 (Mmp3), and (**J**) histidine decarboxylase (Hdc). Values are reported as mean ± standard error of the mean, n = 4–7/group. * indicates *p*-value < 0.05, ** indicates *p*-value < 0.01, and *** indicates *p*-value < 0.001 using three-way ANOVA with Fisher’s least significant difference for multiple comparisons.

RNA-seq changes were supported by real-time qPCR findings of selected genes shown in Figure 4. Proinflammatory interleukin genes, Il1a (*p* = 0.002), Il1b (*p* = 0.004), and Il6 (*p* < 0.001) were significantly different among the groups (Figure 4A–C). There were significant MPL*fat*sex interactions revealing higher mRNA expression in male LFD 2% MPL-fed mice compared to LFD 1% and LFD 0% control mice for Il1a, Il1b, and Il6 (*p* < 0.001). Other genes commonly induced during colitis involving inflammation and host antimicrobial response were also increased by LFD 2% in male mice. Significant MPL*fat*sex interactions showed higher mRNA expression in male LFD 2% MPL-fed mice compared to LFD 1% and LFD 0% control mice for Acod1 (*p* < 0.001), Lcn2 (*p* < 0.001), Cxcl1 (*p* < 0.001), and Hdc (*p* < 0.05) (Figure 4D,G,H,J). In addition, male mice fed LFD 2% had increased Mmp3 expression compared to LFD 0% (*p* = 0.006), HFD 2% (*p* = 0.009), and females fed LFD 2% (*p* = 0.003) (Figure 4I). In contrast, male mice fed MPLs at 1% (*p* = 0.02), and 2% (*p* = 0.06) on HFD had lower Mmp3 expression compared to HFD 0% control. Interestingly, there was a significant MPL*fat interaction for colon intelectin-1 (Itln1) mRNA expression, with mice fed HFD 2% having higher Itln1 expression than mice fed LFD 2%, HFD 1%, and HFD 0% (*p* < 0.001) (Figure 4F).

### 3.3. MPLs and Male Sex Increases Fecal Microbial Diversity but Does Not Alter Microbial Phyla Composition

**Figure 5:**
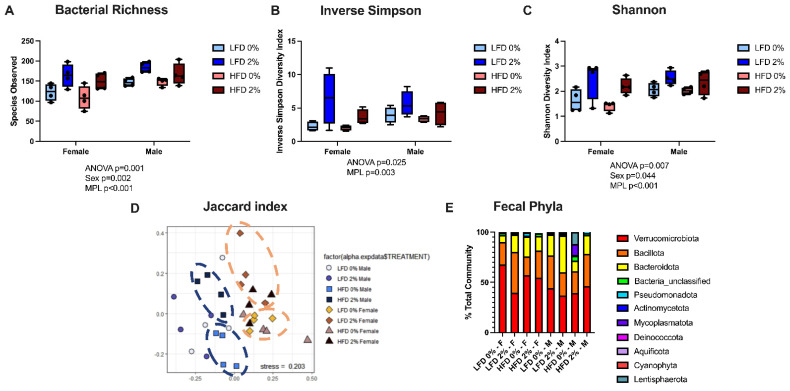
MPLs increase fecal microbial diversity. Cecal feces were collected and analyzed using 16S rRNA sequencing (n = 4/group). Alpha diversity (**A**–**C**), beta diversity (**D**), and relative abundance at phylum (**E**) are shown. Diversity measures were analyzed via nonmetric multidimensional scaling and taxa comparisons were analyzed via three-way ANOVA with Fisher’s Least Significant Difference for multiple comparisons.

DSS treatment is known to significantly worsen gut microbial diversity in mice [22]. The addition of 2% MPLs increased fecal bacterial richness, via an increase in the number of species, and Shannon Index vs. 0% controls (*p* < 0.001) (Figure 5A,C). Inverse Simpson was also increased by 2% MPLs (*p* = 0.003) (Figure 5B). MPLs did not affect Bray-Curtis or Theta YC measures of beta diversity (data not shown), but the Jaccard index was significantly different between HFD 2% vs. 0% males and LFD 2% vs. 0% females (Figure 5D). Males had higher Shannon index (*p* = 0.009) and species observed (*p* = 0.002). In addition, the Jaccard index, Bray-Curtis, Shannon, and inverse Simpson indexes were significantly different in control groups between males and females, indicating different microbial communities (Figure 5B–D). Thus, the presence of MPLs and the male sex independently increased both alpha and beta measures of microbial diversity. In contrast, MPLs did not alter the gut microbial composition at the phylum level except for unclassified bacteria (*p* = 0.034) (Figure 5E). There was a fat*sex interaction (*p* = 0.005) where males fed HFD had increased unclassified bacteria compared to both male mice fed LFD and female mice fed HFD (*p* = 0.002).

### 3.4. MPLs Increase Fecal Ceramides and Sphingomyelins

**Figure 6:**
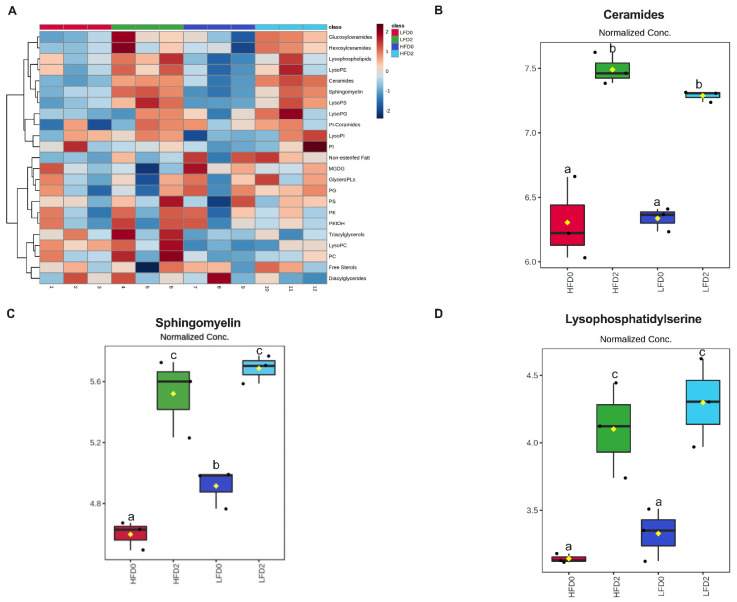
MPLs increase fecal phospholipid and sphingolipid metabolites in male mice. A heatmap of the sum of lipid classes (**A**) and total ceramides (**B**), sphingomyelin (**C**), and lysophosphatidylserines (**D**). Data were normalized using log transformation and were analyzed using a one-way ANOVA with false discovery rate FDR q-value < 0.05 (n = 3/group). Values with unlike letters indicate differences at *p* < 0.05 using post-hoc comparisons. Abbreviations: LysoPE, lysophosphatidylethanolamine; LysoPS, lysophosphatidylserines; LysoPG, lysophosphatidylglycerol; PI-Ceramides, phosphatidylinositol-cermamides; LysoPI, lysophosphatidylinositol; PI, phosphatidylinositol; MGDG; Monogalactosyldiacylglycerols; GlyeroPLs, glycerophospholipids; PG, phosphatidylglycerol; PS, phosphatidylserine; PE, phosphatidylethanolamine; PEtOH, phosphatidylethanol; LysoPC, lysophosphatidylcholine; PC, phosphatidylcholine.

**Figure 7:**
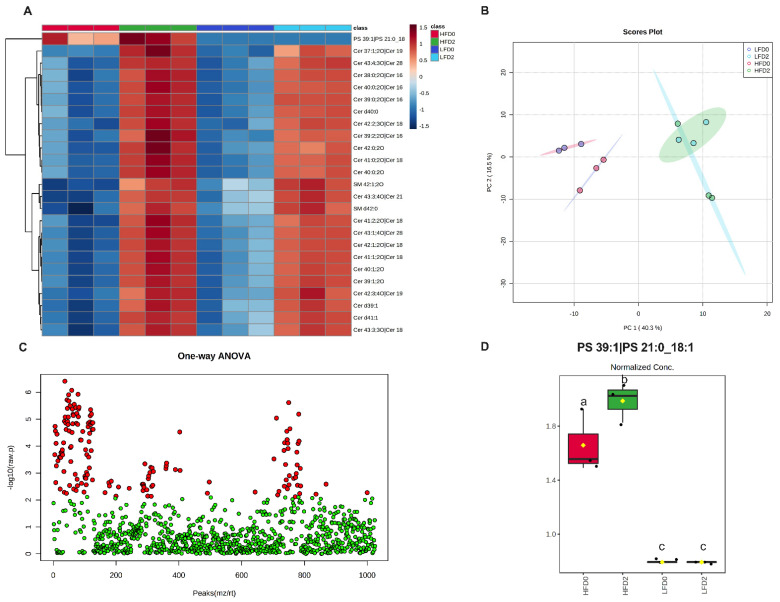
MPLs increase fecal ceramide and sphingolipid species in male mice. A heat map of the top 25 lipid species (**A**), PCA plot (**B**), one-way ANOVA plot (**C**), and phosphatidylserine (PS) 39:1 species (**D**) are shown. Data were normalized using log transformation and were analyzed using a one-way ANOVA with false discovery rate (FDR) q-value < 0.05 (n = 3/group). Values with unlike letters indicate differences at *p* < 0.05 using post-hoc comparisons. Abbreviations: Cer, Ceramide; SM, sphingomyelin.

Fecal lipidomics was analyzed only in male mice to determine whether differences in lipidomics could explain differences seen in disease activity between the MPL-fed male mice. There were 1029 lipids identified in fecal samples of male mice. The most abundant lipid species included behenic acid, lignoceric acid, N-(tricosanoyl)-sphing-4-enine (Cer d41:1), N-docosanoylsphingosine (Cer d40:1), and N-palmitoyl-sphingosine (Cer d34:1). Figure 6A shows a heat map of the fecal lipid classes. Of the lipid classes, total fecal ceramides, sphingomyelin, and lysophosphatidylserines were significantly different between groups (Figure 6B–D and Table S3). Total fecal ceramides were increased in both LFD 2% and HFD 2% compared to their respective controls (Figure 6B). Similarly, total fecal sphingomyelin increased with MPL consumption in both diets but did not differ between the MPL-fed groups (Figure 6C). Lastly, total lysophosphatidylserines were increased in the MPL-fed groups (Figure 6D). A one-way ANOVA revealed 155 significantly different lipid species (Figure 7C and Table S4). A PCA plot and a heat map of the top 25 lipids are shown in Figure 7A,B. MPL supplementation significantly increased long-chain and very-long-chain ceramides and sphingomyelin species in both LFD and HFD. Interestingly, fecal phosphatidylserine (PS) 39:1|(PS 21:0_18:1) was increased in HFD 2% mice compared to HFD controls (Figure 7D), but this lipid was nearly absent in feces of LFD-fed mice.

## 4. Discussion

Therapeutic targets are limited for those with IBD; thus, it is critical to identify strategies to mitigate symptoms and disease progression. We have previously reported that dietary polar lipids, including sphingomyelin from egg and milk, have anti-inflammatory properties in several mouse models and can improve fecal microbial diversity [11,13,14,15]. However, some researchers have indicated that dietary egg sphingomyelin can promote apoptosis and cytokine signaling in a colitis mouse model [23]. Anhydrous milk fat, a rich source of neutral lipids, has been shown to promote colitis and inflammation in mice [9]. Therefore, we sought to investigate the effect of varying compositions of milk fat and MPLs on colitis disease severity and the fecal microbiota and lipid composition. Male and female mice were included to identify sex-dependent responses. In this study, the effects of MPLs on disease activity were significantly different between LFD and HFD-fed animals. In HFD-fed mice, DAI scores were lower in the MPL-fed groups, while in the LFD-fed male mice the DAI scores were higher in the 2% MPL-fed group, compared to their respective control groups (0% MPL).

Previous literature has mostly supported the anti-inflammatory properties of dietary phospholipids and sphingolipids. Mazzei et al. examined the effects of 0.1% pure milk sphingomyelin in AIN-76A diet in wild-type and *Pparg*^-/-^ mice fed for a week and then injected mice with azoxymethane and given 2% (w/v) DSS a week later to induce colon inflammation [10]. Milk sphingomyelin suppressed DAI in both genotypes during the recovery period and improved survival rate, while being more protective against DAI in the wild-type mice but more protective against mortality in *Pparg*^-/-^ mice [10]. Milk sphingomyelin supplementation reduced F4/80+ macrophages in mesenteric lymph nodes and there was a trend for reduced CD4+ T cells [10]. In addition, sphingomyelin recovered crypt-like colonic structures but did not prevent a massive influx of immune cells [10]. In colons, the authors reported an upregulation of Th2 differentiation pathways and regulatory T cell-related genes in a PPARγ-dependent manner and the suppression of genes associated with apoptosis and inflammatory signaling [10]. Similar protective effects have been reported for dietary phosphatidylcholine. Recently, Li et al. compared the effects of 30 mg/kg soybean phosphatidylcholine and egg sphingomyelin with AIN-93 diet on 3% DSS-induced colitis in institute of cancer research (ICR) mice for 15 days. The investigators found that while dietary egg sphingomyelin improved disease activity, feeding soybean phosphatidylcholine had a stronger anti-inflammatory effect [24]. In contrast, Fischbeck et al. showed that oral gavage of egg sphingomyelin (4 mg/day for seven days) promoted inflammation and induced apoptosis, resulting in greater mucosal damage in DSS-induced murine colitis [23]. They found increased levels of sphingomyelin and ceramide in the feces and intestinal epithelial cells of mice fed egg sphingomyelin [23]. It is difficult to compare results across studies due to differences in food source of polar lipids, mouse genotype, and DSS colitis protocols. Another key difference between the diets used in the studies discussed above and the present study is the fat content and source. Mazzei et al. and Li et al. used low-fat diets containing higher amounts of mono- and poly-unsaturated fats, while anhydrous milk fat has a higher content of saturated fats [25]. Fischbeck et al. used water for the oral gavage of egg sphingomyelin, which may influence its properties due to a lack of interaction with other food components, such as fat. Milk fat contains short-chain free fatty acids, aldehydes, ketones, and lactones [26]. Milk sphingomyelin has been shown to be a potent inhibitor of fat absorption [27], and, thus, in mice fed a HFD there could be a greater proportion of intact sphingomyelin entering the colon. In contrast, in mice fed the low-fat diet, the SM could be getting metabolized to ceramide, which can exert pro-inflammatory effects [28]. Therefore, the anti-inflammatory effects of MPLs could be dependent on the base dietary fat content and source.

To better understand the mechanism of how milk fat and MPL influence colitis, we examined the colon transcriptome via RNA-seq and confirmed a selection of genes via real-time qRT-PCR. In the present study, the colons of male mice fed HFD with 2% MPLs significantly reduced gene expression related to inflammatory signaling and cytokine interactions compared to HFD control. MPLs have previously been reported to have anti-inflammatory properties in different mouse models. Furuya et al. found that dietary sphingomyelin prevented the increase in inflammation in DSS-treated mice potentially by secreting IgA into the large intestine [29]. We have previously reported that lipopolysaccharide-activated macrophages treated with 0.8 μg/mL milk sphingomyelin had reduced Tnf and Ccl2 mRNA expression compared to controls [12]. In addition, high-fat and cholesterol diet-fed mice supplemented with 0.1% (w/w) milk sphingomyelin for 10 weeks decreased serum TNFα, IL-6, and CCL4 concentrations [12]. Furthermore, feeding 2% (w/w) MPLs in Ldlr-/- mice for 14 weeks attenuated inflammatory signaling caused by HFD [11]. However, contrary to most of the literature, in the current study MPLs increased the inflammatory gene expression in LFD, specifically in males. Male mice fed LFD 2% had 118 upregulated DEG that were associated with inflammation and cytokine signaling. There is some evidence that milk sphingomyelin can increase cytokine expression. In line with our findings, a PCR array of colons from DSS-treated mice revealed an upregulation of cytokines and chemokine receptors with milk sphingomyelin supplementation [10]. Mazzei et al. hypothesized that sphingomyelin can induce inflammation with a parallel anti-inflammatory response [10]. Thus, dietary sphingomyelin may act to reduce inflammation and promote an anti-inflammatory environment in HFD-fed mice but increase cytokines in LFD-fed mice. Mazzei et al. discovered that sphingomyelin can act dependently and independently of PPARγ [10]; however, colon transcriptomics in the current study did not reveal differences in PPARγ targets between the groups.

Further analysis of gene expression from RNA-seq data with real-time qRT-PCR revealed alterations in the colonic expression of genes involved in host antimicrobial response and neutrophil activation. Antimicrobial peptides are produced in response to colonic injury, especially colitis [30]. LFD 2% male mice had higher expression of Acod1, Lcn2, and S100a9, which are upregulated in colitis and inflammatory bowel disease [31]. Hdc was also significantly higher in LFD MPL groups compared to its respective control group and HFD counterpart. Hdc is crucial for the formation of histamine in neutrophils whose circulating levels are higher in IBD patients [32]. In contrast, MPLs in HFD-augmented-gene expression of antimicrobial peptide Itln1 can inhibit lipopolysaccharide-induced cytokine production and phagocytosis in macrophages via the inhibition of NF-κB [33,34]. In the current study, MPL feeding increased anti-microbial gene expression in HFD without increasing inflammation; however, both increases in anti-microbial gene expression and inflammation were seen with MPL feeding of male mice on LFD. Overall, compared to mice-fed-control diets without added MPLs, the supplementation of LFD with 2% MPL exacerbated colon inflammatory gene expression in male mice, while to a lesser extent, the supplementation of HFD with 2% MPL attenuated colon inflammation in male mice. Thus, our findings suggest the base diet milk fat content and biological sex influence the anti-inflammatory effect of MPLs in the DSS model of colitis.

In IBD, colonic motor activity and motility affect diarrhea and pain, hallmark features of the disease [35]. Low-motor activity can promote inflammation and impair the contractility of smooth muscle cells [35,36]. Decreased intracellular Ca^2+^ stores and increased IL-1β can inhibit smooth muscle cell contraction in ulcerative colitis [36]. Colonic Il1b mRNA expression was increased in LFD 2%-fed male mice. In addition, RNA-seq revealed a downregulation of genes associated with calcium and cyclic AMP (cAMP) signaling two pathways that can downregulate cAMP-responsive element-binding protein (CREB) signaling [37]. CREB mediates the expression of tight junction zonula occludens-1 and suppresses NF-κB signaling to protect intestinal barrier function [38]. Colon RNA-seq also showed decreased mRNA related to muscle contraction and regulation of muscle contraction processes, in line with decreased Ca^2+^ stores and increased Il1b mRNA expression. MPLs may contribute to colitis-induced impaired colonic motility and contraction in male mice fed LFD. This is the first study to indicate that MPLs may play a role in colonic muscle contraction in LFD-fed male mice in a model of chemically induced colitis.

To identify other potential differences between groups, lipidomics of fecal samples were performed on male mice. It has been reported that sphingolipids are the most differentially abundant fecal metabolite in IBD patients compared to controls, with ceramides and sphingosine among the top 13 increased metabolites in UC patients [16]. A total of 2% MPL supplementation increased total fecal ceramides and sphingomyelin, which is to be expected from the increased dietary sphingolipids with MPL feeding. More specifically, long-chain and very-long-chain ceramides and sphingomyelins were increased, which is also expected since milk sphingolipids contain very-long-chain fatty acids. The most dramatic change in fecal lipidomics between the groups was in phosphatidylserine (PS) 39:1|(PS 21:0_18:1), which was only present in HFD-fed groups and was increased with 2% MPL supplementation.

Lastly, we employed 16S rRNA sequencing to analyze the fecal microbiota composition and to identify the effects of MPLs on bacterial diversity. We observed that 2% of MPLs increased species richness and diversity measures in both LFD and HFD. Previous research has supported that MPLs can improve microbial diversity in HFD-fed animals. Li et al. found that 30 mg/kg of sphingomyelin in mice given DSS restored alpha diversity of the gut microbiome compared to control using the Chao and Ace Indices [24]. Millar et al. found that 2% MPLs in HFD-fed mice modulated gut microbiota via significantly different Jaccard and Bray-Curtis beta diversity indices [11]. Similarly, 400 mg/kg polar-lipid enriched milk fat globule membrane in pregnant and lactating female Sprague Dawley rats enhanced alpha diversity measures of gut microbiota including Ace, Chao, Simpson, and Shannon indexes after 8 weeks [39]. As LFD-fed male mice supplemented with 2% MPL in the current study had both increased fecal bacterial diversity and increased colon inflammation compared to LFD control diet, our data suggest that the increased fecal bacterial diversity seen with MPL feeding in mice may not always coincide with reductions in colon inflammation.

## 5. Conclusions

In summary, our data support existing research that dietary MPLs have anti-inflammatory properties and can improve fecal microbial diversity when supplemented in HFD-fed animals. However, this study is unique in that we show differences in the anti-inflammatory properties of MPLs based on fat content of the diet and sex of the mice. This supports the notion that bioactive properties of dairy products can depend on the food matrices [40].

## Figures and Tables

**Table 1:** Diet Composition.

Diet Component (g per 1 kg)	LFD 0%	LFD 1%	LFD 2%	HFD 0%	HFD 1%	HFD 2%
Casein	80	80	80	80	80	80
L-Cystine	3	3	3	3	3	3
Sucrose	200	200	200	200	200	200
Corn Starch	175	172.5	170	20	17.5	15
Lactose	21.35	10.68	0	21.35	10.68	0
Anhydrous Milkfat	52	26	0	207	181	155
Soybean Oil	20	20	20	20	20	20
Cellulose	50	50	50	50	50	50
Mineral Mix, AIN-93G-MX (94046)	43	43	43	43	43	43
Vitamin Mix, AIN-93-VX (94047)	19	19	19	19	19	19
Choline Bitartrate	3	3	3	3	3	3
TBHQ, antioxidant	0.04	0.04	0.04	0.04	0.04	0.04
Cholesterol	1.5	1.3	1.12	1.5	1.3	1.12
Skim milk powder—Dyets 403150	332	166	0	332	166	0
Beta serum powder—Tatua BSP2	0	205.5	411	0	205.5	411
Calcium Carbonate (40% calcium by wt)	0	0.68	1.36	0	0.68	1.36
Potassium Chloride (53% potassium by wt)	0	0.19	0.38	0	0.19	0.38
Sodium Chloride (40% sodium by wt)	0	0.25	0.51	0	0.25	0.51

Abbreviation: TBHQ, tert-butylhydroquinone; wt, weight.

**Table 2:** Diet Composition by Macronutrient.

Diet Component	LFD 0%	LFD 1%	LFD 2%	HFD 0%	HFD 1%	HFD 2%
**Total Protein (g/kg)**	200.2	200.3	200.4	200.2	200.3	200.4
**Total Carbohydrate (g/kg)**	568.7	566.5	564.0	414.0	411.5	409.0
**Total Fat (g/kg)**	74.7	76.3	78.0	229.7	231.3	233.0
**Total Lactose (g/kg)**	194.0	194.0	194.0	194.0	194.0	194.0
**Total Cholesterol (g/kg)**	1.6	1.68	1.68	2.06	2.06	2.06
**Total Phospholipid (g/kg)**	0.5	10.3	20.1	0.5	10.3	20.1
**% kcal from Protein**	21.4	21.3	21.3	17.7	17.7	17.7
**% kcal from Carbohydrate**	60.7	60.4	60.0	36.6	36.3	36.1
**% kcal from Fat**	17.9	18.3	18.7	45.7	46	46.2
**Calorie Density (kcal/g)**	3.75	3.75	3.75	4.52	4.52	4.52

**Table 3:** Body, Tissue Weights, and Food Intake.

							*p*-Value (Three-Way ANOVA)			
	LFD 0%	LFD 1%	LFD 2%	HFD 0%	HFD 1%	HFD 2%	MPL	Fat	Sex	Interaction
End Body Weight (g)										
Female	21.52 ± 1.30	21.02 ± 1.46	21.27 ± 1.11	21.24 ± 1.49	22.87 ± 2.60	21.48 ± 1.56	n.s.	<0.001	<0.001	MPL*Fat (0.018) MPL*Sex (0.009) Fat*Sex*MPL (0.036)
Male	24.48 ± 1.60	25.48 ± 1.29	24.69 ± 1.88	26.65 ± 1.89	27.53 ± 1.29	27.87 ± 1.72				
Total Food Intake (g)										
Female	300.4 ± 24.7	331.6 ± 41.2	304.5 ± 59.2	295.9 ± 37.0	387.3 ± 53.6	362.0 ± 98.2	n.s.	n.s.	n.s.	n.s.
Male	307.9 ± 7.3	325.4 ± 55.3	292.7 ± 10.6	354.5 ± 149.8	322.0 ± 25.9	346.8 ± 108.3				
Spleen (g)										
Female	0.11 ± 0.02	0.13 ± 0.04	0.11 ± 0.02	0.11 ± 0.02	0.11 ± 0.02	0.09 ± 0.01	n.s.	n.s.	n.s.	n.s.
Male	0.09 ± 0.03	0.08 ± 0.01	0.09 ± 0.04	0.10 ± 0.04	0.17 ± 0.25	0.09 ± 0.02				
Liver (g)										
Female	0.97 ± 0.11	0.87 ± 0.12	0.89 ± 0.12	0.88 ± 0.10	0.85 ± 0.12	0.82 ± 0.07	0.046	n.s.	<0.001	n.s.
Male	1.06 ± 0.19	0.92± 0.09	0.98 ± 0.14	0.97 ± 0.17	0.99 ± 0.11	0.97 ± 0.12				
Adipose (g)										
Female	0.37 ± 0.09	0.22 ± 0.13	0.22 ± 0.11	0.33 ± 0.11	0.41 ± 0.24	0.30 ± 0.17	n.s.	<0.001	<0.001	Fat*Sex (0.008)
Male	0.48 ± 0.15	0.60 ± 0.11	0.37 ± 0.19	0.65 ± 0.35	0.83 ± 0.42	0.87 ± 0.36				
Colon Length (cm)										
Female	6.06 ± 0.60	6.14 ± 0.62	6.60 ± 0.84	6.67 ± 0.69	6.43 ± 0.64	6.59 ± 0.87	n.s.	n.s.	n.s.	n.s.
Male	6.52 ± 0.93	6.60 ± 1.10	6.15 ± 1.05	6.95 ± 0.69	6.73 ± 0.90	7.18 ± 0.84				
Colon Weight (g)										
Female	0.18 ± 0.02	0.18 ± 0.02	0.18± 0.02	0.16 ± 0.03	0.18 ± 0.02	0.15 ± 0.02	0.001	0.021	n.s.	MPL*Fat (0.031) MPL*Sex (0.002)
Male	0.16 ± 0.02	0.15 ± 0.02	0.21 ± 0.04	0.17 ± 0.04	0.16 ± 0.02	0.16 ± 0.04				

Values reported as mean ± standard deviation of the values for n = 10 / group. *P*-values are from Three-way ANOVA with milk polar lipids (0%, 1%, 2% MPL), fat (LFD or HFD), and sex (male or female) as factors. * indicates an interaction between factors. Abbreviations: MPL, milk polar lipids; n.s., not significant.

## Data Availability

The datasets used and/or analyzed during the current study are available on request from the corresponding author.

## Supplementary Materials

The following supporting information can be downloaded at: https://www.mdpi.com/article/10.3390/nu14235145/s1, Figure S1: MPLs protect against bleeding while MPLS exacerbate weight loss and stool consistency in LFD; Table S1: PCR Primer Sequences; Table S2: Colon RNAseq DEG Results; Table S3: Lipid Classes Lipidomics ANOVA; Table S4: Species Specific Lipidomics ANOVA.
