# Supplementary material for: Effects of Milk Polar Lipids on DSS-Induced Colitis Severity Are Dependent on Dietary Fat Content
Source: Nutrients. 2022 Dec 3;14(23):5145. doi: 10.3390/nu14235145 (PMC9738862; doi:10.3390/nu14235145)
Supplement: Supplementary file 1 [file nutrients-14-05145-s001.zip › Supplemental Table S1. PCR Primer Sequences.pdf]

Table S1. PCR Primer Sequences

Gene	Forward (5' → 3')	Reverse (5' → 3')
Acod1	AGTTTTCTGGCCTCGACCTG	AGAGGGAGGGTGGAATCTCT
Actb	CGATGCCCTGAGGCTCTTT	TGGATGCCACAGGATTCCA
Arg1	TTCTCAAAAGGACAGCCTCG	CAGACCGTGGGTTCTTCACA
Cxcl1	TGCACCCAAACCGAAGTCAT	CTCCGGTTACTTGGGGACACC
Cxcl5	TGCCCTACGGTGGAAGTCAT	AGCTTTCTTTTTGTCACTGCCC
Gapdh	TGTGTCCGTCGTGGATCTGA	CCTGCTTCACCACCTTCTTGAT
Hdc	ATGGAGCCCTGTGAATACCG	TGTAATCCACCATCTCTTTCCCTCT
Il1a	GTCGGGAGGAGACGACTCTAA	GTTTCTGGCAACTCCTTCAGC
Il1b	GTCACAAGAAACCATGGCACAT	GCCCATCAGAGGCAAGGA
Il6	CTGCAAGAGACTTCCATCCAGTT	AGGGAAGGCCGTGGTTGT
Itln1	ATCATGGTTGCTACCAGAGGTTG	CAGGAAATAGAGACCATCTTGTGC
Lcn2	CCACCACGGACTACAACCAG	AGCTCCTTGGTTCTTCCATACA
Mmp3	AGTCCCTCTATGGAACTCCC	AGATTTGCGCCAAAAGTGCC
Rplp0	CCTGAAGTGCTCGACATCAC	CCACAGACAATGCCAGGAC
S100a8	GTCCTCAGTTTGTGCAGAATATAAA	GGCCAGAAGCTCTGCTACTC
S100a9	TGGGCTTACACTGCTCTTACC	GGTTATGCTGCGCTCCATCT
Tnf	CTCTTCTGTCTACTGAAC	AAGATGATCTGAGTGTGA
