# Supplementary material for: Effects of Milk Polar Lipids on DSS-Induced Colitis Severity Are Dependent on Dietary Fat Content
Source: Nutrients. 2022 Dec 3;14(23):5145. doi: 10.3390/nu14235145 (PMC9738862; doi:10.3390/nu14235145)
Supplement: Supplementary file 1 [file nutrients-14-05145-s001.zip › Supplemental Table S3. Lipid Classes Lipidomics ANOVA.pdf]

Table S3. Sum of Lipid Classes ANOVA

**Lipid** Class	**f.value**	**p.value**	**-LOG10(p)**	**FDR**	**Fisher's LSD**
**Ceramides**	36.63	5.07E-05	4.2946	0.0010312	HFD2 - HFD0; LFD2 - HFD0; HFD2 - LFD0; LFD2 - LFD0
**Sphingomyelin**	31.38	8.9666E-05	4.0474	0.0010312	HFD2 - HFD0; LFD0 - HFD0; LFD2 - HFD0; HFD2 - LFD0; LFD2 - LFD0
**Lysophosphatidylserines**	14.412	0.0013682	2.8639	0.010489	HFD2 - HFD0; LFD2 - HFD0; HFD2 - LFD0; LFD2 - LFD0
