# Supplementary material for: Effects of Milk Polar Lipids on DSS-Induced Colitis Severity Are Dependent on Dietary Fat Content
Source: Nutrients. 2022 Dec 3;14(23):5145. doi: 10.3390/nu14235145 (PMC9738862; doi:10.3390/nu14235145)
Supplement: Supplementary file 1 [file nutrients-14-05145-s001.zip › Supplemental Table S4. Species specific Lipidomics ANOVA.pdf]

**Table S4. Top 50 Features from Lipidomics Analysis**

| Lipid Species | f.value | p.value | -LOG10(p) | FDR | Fisher's LSD |
|---|---|---|---|---|---|
| **Cer 37:1;2O\|Cer 19:1;2O/18:0** | 130.93 | 3.88E-07 | 6.4117 | 0.00030366 | HFD2 - HFD0; LFD2 - HFD0; HFD2 - LFD0; HFD2 - LFD2; LFD2 - LFD0 |
| **Cer 41:0;2O\|Cer 18:0;2O/23:0** | 107.29 | 8.43E-07 | 6.0742 | 0.00030366 | HFD2 - HFD0; LFD2 - HFD0; HFD2 - LFD0; LFD2 - LFD0 |
| **Cer 43:1;4O\|Cer 28:0;3O/15:1;(2OH)** | 98.288 | 1.19E-06 | 5.9262 | 0.00030366 | HFD2 - HFD0; LFD2 - HFD0; HFD2 - LFD0; LFD2 - LFD0 |
| **Cer 40:0;2O** | 97.173 | 1.24E-06 | 5.9069 | 0.00030366 | HFD2 - HFD0; LFD2 - HFD0; HFD2 - LFD0; LFD2 - LFD0 |
| **Cer 39:0;2O\|Cer 16:0;2O/23:0** | 86.74 | 1.92E-06 | 5.7157 | 0.00030366 | HFD2 - HFD0; LFD2 - HFD0; HFD2 - LFD0; LFD2 - LFD0 |
| **Cer 40:0;2O\|Cer 16:0;2O/24:0** | 84.701 | 2.11E-06 | 5.6758 | 0.00030366 | HFD2 - HFD0; LFD2 - HFD0; HFD2 - LFD0; LFD2 - LFD0 |
| **SM 42:1;2O** | 81.79 | 2.41E-06 | 5.6171 | 0.00030366 | HFD2 - HFD0; LFD0 - HFD0; LFD2 - HFD0; HFD2 - LFD0; LFD2 - LFD0 |
| **Cer 42:1;2O\|Cer 18:1;2O/24:0** | 78.619 | 2.81E-06 | 5.5509 | 0.00030366 | HFD2 - HFD0; LFD2 - HFD0; HFD2 - LFD0; LFD2 - LFD0 |
| **Cer 40:1;2O** | 76.223 | 3.17E-06 | 5.4991 | 0.00030366 | HFD2 - HFD0; LFD2 - HFD0; HFD2 - LFD0; LFD2 - LFD0 |
| **Cer 43:4;3O\|Cer 28:2;3O/15:2** | 73.847 | 3.58E-06 | 5.4461 | 0.00030366 | HFD2 - HFD0; LFD2 - HFD0; HFD2 - LFD0; LFD2 - LFD0 |
| **Cer 42:2;3O\|Cer 18:1;2O/24:1;O** | 73.096 | 3.72E-06 | 5.4291 | 0.00030366 | HFD2 - HFD0; LFD2 - HFD0; HFD2 - LFD0; LFD2 - LFD0 |
| **Cer 39:1;2O** | 71.995 | 3.95E-06 | 5.4037 | 0.00030366 | HFD2 - HFD0; LFD2 - HFD0; HFD2 - LFD0; LFD2 - LFD0 |

| | | | | | |
|---|---|---|---|---|---|
| **Cer 43:3;4O\|Cer 21:3;3O/22:0;(2OH)** | 71.059 | 4.15E-06 | 5.3819 | 0.00030366 | HFD2 - HFD0; LFD0 - HFD0; LFD2 - HFD0; HFD2 - LFD0; LFD2 - LFD0 |
| **Cer 41:1;2O\|Cer 18:1;2O/23:0** | 70.717 | 4.23E-06 | 5.3738 | 0.00030366 | HFD2 - HFD0; LFD2 - HFD0; HFD2 - LFD0; LFD2 - LFD0 |
| **Cer d40:0** | 69.807 | 4.44E-06 | 5.3522 | 0.00030366 | HFD2 - HFD0; LFD2 - HFD0; HFD2 - LFD0; LFD2 - LFD0 |
| **Cer 42:3;4O\|Cer 19:3;3O/23:0;(2OH)** | 64.235 | 6.11E-06 | 5.2138 | 0.00035448 | HFD2 - HFD0; LFD2 - HFD0; HFD2 - LFD0; LFD2 - LFD0 |
| **Cer d41:1** | 63.346 | 6.45E-06 | 5.1907 | 0.00035448 | HFD2 - HFD0; LFD2 - HFD0; HFD2 - LFD0; LFD2 - LFD0 |
| **SM d42:0** | 63.256 | 6.48E-06 | 5.1883 | 0.00035448 | HFD2 - HFD0; LFD0 - HFD0; LFD2 - HFD0; HFD2 - LFD0; LFD2 - LFD0 |
| **Cer 41:2;2O\|Cer 18:1;2O/23:1** | 62.987 | 6.59E-06 | 5.1812 | 0.00035448 | HFD2 - HFD0; LFD2 - HFD0; HFD2 - LFD0; LFD2 - LFD0 |
| **Cer 42:0;2O** | 61.304 | 7.31E-06 | 5.1363 | 0.00035448 | HFD2 - HFD0; LFD2 - HFD0; HFD2 - LFD0; LFD2 - LFD0 |
| **Cer d39:1** | 61.042 | 7.43E-06 | 5.1292 | 0.00035448 | HFD2 - HFD0; LFD2 - HFD0; HFD2 - LFD0; LFD2 - LFD0 |
| **Cer 43:3;3O\|Cer 18:2;2O/25:1;O** | 59.715 | 8.08E-06 | 5.0928 | 0.00035448 | HFD2 - HFD0; LFD2 - HFD0; HFD2 - LFD0; LFD2 - LFD0 |
| **Cer 38:0;2O\|Cer 16:0;2O/22:0** | 59.337 | 8.27E-06 | 5.0823 | 0.00035448 | HFD2 - HFD0; LFD2 - HFD0; HFD2 - LFD0; LFD2 - LFD0 |
| **Cer 39:2;2O\|Cer 16:1;2O/23:1** | 59.288 | 8.30E-06 | 5.0809 | 0.00035448 | HFD2 - HFD0; LFD2 - HFD0; HFD2 - LFD0; LFD2 - LFD0 |
| **PS 39:1\|PS 21:0_18:1** | 57.838 | 9.12E-06 | 5.04 | 0.00037393 | HFD2 - HFD0; HFD0 - LFD0; HFD0 - LFD2; HFD2 - LFD0; HFD2 - LFD2 |

| | | | | | |
|---|---|---|---|---|---|
| **Cer 37:0;2O\|Cer 16:0;2O/21:0** | 53.876 | 1.19E-05 | 4.9229 | 0.00043983 | HFD2 - HFD0; LFD2 - HFD0; HFD2 - LFD0; LFD2 - LFD0 |
| **Cer 39:1;2O\|Cer 16:1;2O/23:0** | 53.344 | 1.24E-05 | 4.9065 | 0.00043983 | HFD2 - HFD0; LFD2 - HFD0; HFD2 - LFD0; LFD2 - LFD0 |
| **Cer d38:1** | 53.071 | 1.26E-05 | 4.8981 | 0.00043983 | HFD2 - HFD0; LFD2 - HFD0; HFD2 - LFD0; LFD2 - LFD0 |
| **Cer d43:1** | 52.124 | 1.35E-05 | 4.8685 | 0.00043983 | HFD2 - HFD0; LFD2 - HFD0; HFD2 - LFD0; LFD2 - LFD0 |
| **Cer d42:0** | 52.11 | 1.36E-05 | 4.868 | 0.00043983 | HFD2 - HFD0; LFD2 - HFD0; HFD2 - LFD0; LFD2 - LFD0 |
| **Cer 38:1;2O\|Cer 18:1;2O/20:0** | 51.724 | 1.39E-05 | 4.8558 | 0.00043983 | HFD2 - HFD0; LFD2 - HFD0; HFD2 - LFD0; LFD2 - LFD0 |
| **Cer d33:1** | 51.672 | 1.40E-05 | 4.8542 | 0.00043983 | HFD2 - HFD0; LFD2 - HFD0; HFD2 - LFD0; HFD2 - LFD2; LFD2 - LFD0 |
| **Cer 42:3;3O\|Cer 18:1;2O/24:2;O** | 51.444 | 1.42E-05 | 4.8469 | 0.00043983 | HFD2 - HFD0; LFD2 - HFD0; HFD2 - LFD0; LFD2 - LFD0 |
| **Cer 41:1;3O\|Cer 18:1;2O/23:0;(2OH)** | 50.947 | 1.48E-05 | 4.831 | 0.00043983 | HFD2 - HFD0; LFD2 - HFD0; HFD2 - LFD0; LFD2 - LFD0 |
| **Cer 43:1;2O** | 50.217 | 1.56E-05 | 4.8073 | 0.00043983 | HFD2 - HFD0; LFD2 - HFD0; HFD2 - LFD0; LFD2 - LFD0 |
| **Cer d40:1** | 50.12 | 1.57E-05 | 4.8041 | 0.00043983 | HFD2 - HFD0; LFD2 - HFD0; HFD2 - LFD0; LFD2 - LFD0 |
| **Cer 39:1;4O** | 49.972 | 1.59E-05 | 4.7992 | 0.00043983 | HFD2 - HFD0; LFD2 - HFD0; HFD2 - LFD0; LFD2 - LFD0 |
| **Cer 30:0;2O** | 48.016 | 1.85E-05 | 4.7338 | 0.00049789 | HFD2 - HFD0; HFD0 - LFD0; LFD2 - HFD0; HFD2 - LFD0; HFD2 - LFD2; LFD2 - LFD0 |

| | | | | | |
|---|---|---|---|---|---|
| **Cer d32:1** | 47.604 | 1.91E-05 | 4.7197 | 0.00050113 | HFD2 - HFD0; LFD2 - HFD0; HFD2 - LFD0; HFD2 - LFD2; LFD2 - LFD0 |
| **SM 44:1;2O** | 45.46 | 2.27E-05 | 4.6444 | 0.00057612 | HFD2 - HFD0; LFD0 - HFD0; LFD2 - HFD0; HFD2 - LFD0; LFD2 - LFD0 |
| **Cer d44:1** | 44.874 | 2.38E-05 | 4.6233 | 0.00057612 | HFD2 - HFD0; LFD2 - HFD0; HFD2 - LFD0; LFD2 - LFD0 |
| **Cer 38:2;2O** | 44.733 | 2.41E-05 | 4.6181 | 0.00057612 | HFD2 - HFD0; HFD0 - LFD0; LFD2 - HFD0; HFD2 - LFD0; LFD2 - LFD0 |
| **Cer d42:1** | 44.695 | 2.42E-05 | 4.6167 | 0.00057612 | HFD2 - HFD0; LFD2 - HFD0; HFD2 - LFD0; LFD2 - LFD0 |
| **Cer 40:0;3O** | 43.231 | 2.74E-05 | 4.5625 | 0.00062249 | HFD2 - HFD0; LFD2 - HFD0; HFD2 - LFD0; LFD2 - LFD0 |
| **Cer 30:1;2O** | 43.16 | 2.76E-05 | 4.5599 | 0.00062249 | HFD2 - HFD0; HFD0 - LFD0; HFD2 - LFD0; HFD2 - LFD2; LFD2 - LFD0 |
| **SM 41:0;2O (1)** | 42.944 | 2.81E-05 | 4.5517 | 0.00062249 | HFD2 - HFD0; LFD0 - HFD0; LFD2 - HFD0; HFD2 - LFD0; LFD2 - LFD0 |
| **Cer 48:3;4O\|Cer 33:2;3O(FA 15:0)** | 42.754 | 2.85E-05 | 4.5445 | 0.00062249 | HFD2 - HFD0; LFD2 - HFD0; HFD2 - LFD0; LFD2 - LFD0 |
| **PC 34:0 (1)** | 42.28 | 2.98E-05 | 4.5264 | 0.00063549 | HFD2 - HFD0; LFD0 - HFD0; LFD2 - HFD0; HFD2 - LFD0; LFD2 - LFD0 |
| **Cer 41:0;4O\|Cer 18:0;3O/23:0;(2OH)** | 41.817 | 3.10E-05 | 4.5085 | 0.00064869 | HFD2 - HFD0; LFD2 - HFD0; HFD2 - LFD0; LFD2 - LFD0 |
| **Cer d36:1** | 40.929 | 3.36E-05 | 4.4737 | 0.00068877 | HFD2 - HFD0; HFD0 - LFD0; LFD2 - HFD0; HFD2 - LFD0; HFD2 - LFD2; LFD2 - LFD0 |
